# Real-time video-rate perfusion imaging using multi-exposure laser speckle contrast imaging and machine learning

**DOI:** 10.1117/1.JBO.25.11.116007

**Published:** 2020-11-15

**Authors:** Martin Hultman, Marcus Larsson, Tomas Strömberg, Ingemar Fredriksson

**Affiliations:** a Linköping University, Department of Biomedical Engineering, Linköping, Sweden; b Perimed AB, Järfälla, Stockholm, Sweden

**Keywords:** microcirculation, perfusion, multi-exposure laser speckle contrast imaging, laser speckle contrast imaging, laser speckle contrast analysis, laser Doppler

## Abstract

**Significance:** Multi-exposure laser speckle contrast imaging (MELSCI) estimates microcirculatory blood perfusion more accurately than single-exposure LSCI. However, the technique has been hampered by technical limitations due to massive data throughput requirements and nonlinear inverse search algorithms, limiting it to an offline technique where data must be postprocessed.

**Aim:** To present an MELSCI system capable of continuous acquisition and processing of MELSCI data, enabling real-time video-rate perfusion imaging with high accuracy.

**Approach:** The MELSCI algorithm was implemented in programmable hardware (field programmable gate array) closely interfaced to a high-speed CMOS sensor for real-time calculation. Perfusion images were estimated in real-time from the MELSCI data using an artificial neural network trained on simulated data. The MELSCI perfusion was compared to two existing single-exposure metrics both quantitatively in a controlled phantom experiment and qualitatively *in vivo*.

**Results:** The MELSCI perfusion shows higher signal dynamics compared to both single-exposure metrics, both spatially and temporally where heartbeat-related variations are resolved in much greater detail. The MELSCI perfusion is less susceptible to measurement noise and is more linear with respect to laser Doppler perfusion in the phantom experiment ($R^{2}=0.992$).

**Conclusions:** The presented MELSCI system allows for real-time acquisition and calculation of high-quality perfusion at 15.6 frames per second.

## 1 Introduction

Noninvasive *in vivo* imaging of microcirculatory blood flow or perfusion (average speed times concentration of moving red blood cells) is of interest in several clinical applications, including monitoring of burn wounds^1^^,^^2^ and investigation of peripheral arterial disease,^3^ as well as several others.^4^ One technique that has gained a lot of focus in the last decades is laser speckle contrast imaging [LSCI; sometimes laser speckle contrast analysis (LASCA)] in which tissue is illuminated with a laser and the resulting speckle pattern is detected with a camera. Light that scatters when interacting with moving particles in the tissue will obtain a Doppler frequency shift depending on the velocity of the particles. The interference of light with different frequencies will give rise to fluctuations in the speckle pattern formed on the imaging sensor. The movement of the speckles on the sensor will cause an image blur that increases with exposure time. The local spatial statistics of this blur can be related to the movement of particles in the tissue, as is done in LSCI.^5^

LSCI has been increasingly used over laser Doppler imaging mainly due to its simple setup and fast acquisition and data processing. However, conventional LSCI has drawbacks such as a nonlinear response to perfusion, dependency to static scattering contrast, and a high variability in the presence of noise.^5^^,^^6^ Recent work has gone into calibrating and correcting for these issues to make LSCI a more accurate technique.^7^ Despite this, while it is generally accepted that LSCI is related to microcirculatory perfusion, the relationship is complex and a direct mapping is still not known.^4^^,^^8^ For the older laser Doppler flowmetry (LDF) technique, the relation to microcircular perfusion is better understood, where it is, for example, possible to theoretically show that the perfusion estimate is linearly related to the flow speed.^9^^,^^10^

To address the nonlinearity of LSCI to perfusion, Parthasarathy et al.^11^ proposed a new setup using multiple exposure times. The technique, called multi-exposure laser speckle contrast imaging (MELSCI, sometimes MESI), obtains information about the speckle motion blur at various exposures, enabling more advanced models to be used when estimating perfusion from the contrast.^5^^,^^12^ Several approaches to capture multi-exposure images have been proposed. The initial approach by Parthasarathy et al.^11^ was to use a time-modulated laser to achieve multi-exposure images. By capturing all images with a fixed exposure time but only illuminating the tissue during parts of that time, multi-exposures can be generated. However, a problem with this approach is that images with different exposure times are separated in time since they are captured sequentially. Dragojević et al.^13^ suggested another approach that addressed this drawback. This was based on a high-speed camera, only capturing images at the shortest exposure time required, and in postprocessing adding successive images together to create longer exposure times. These synthetic exposure times are valid if the interframe delay in the camera is negligible. This method is faster and more accurate than previous methods due to all exposure times essentially being captured simultaneously, but it produces immense amounts of data that must be transferred and processed. At the time, this was, therefore, limited to an offline technique. Hence, MELSCI has so far been held back by technical limitations in both the imaging setup and the computation time of the models.

To address this problem of a data and processing bottleneck, we have previously presented a system for synthetic MELSCI,^14^ based on a high-speed 1000-frames per second (fps) 1-megapixel camera directly interfaced to a field programmable gate array (FPGA) performing the multi-exposure contrast algorithm outlined by Dragojević^13^ in real-time. Processed contrast images were sent to a computer, massively reducing the data throughput requirements. This system could produce multi-exposure contrast images at 15.6 fps, using all available frames from the 1000-fps camera, without any loss of data. However, due to insufficient processing power, the system was not able to transfer the processed images to the computer fast enough, and thus could not provide a real-time video-rate output. Furthermore, while the calculation of multi-exposure contrast images was fast enough, at the time there was no multi-exposure perfusion algorithm fast enough to keep up with the continuous stream of contrast images. The model proposed by Parthasarathy et al.^11^ and later refined by Kazmi et al.^15^ requires nonlinear fitting to extract perfusion-related parameters from the multi-exposure contrast. This process must be performed individually in each pixel for full field perfusion images, which is not feasible in real-time with satisfactory frame rates.^6^

We have previously presented a method using machine learning and simulated data from thousands of Monte Carlo tissue models from which we train an artificial neural network (ANN) to translate multi-exposure contrast to laser Doppler perfusion. We showed that this technique not only achieves a model-inference time of <30  ms per image, but also has higher accuracy and less susceptibility to noise than other available models.^6^

The aim of this paper is to present a complete system for continuous real-time video-rate perfusion imaging using both our previous methods in an integrated processing pipeline. We validate the accuracy of the perfusion estimate in a controlled phantom experiment and present results from an occlusion-release provocation of the forearm of a healthy subject. We use these measurements to compare both the quantitative and qualitative similarities and differences between our perfusion estimate and two common single-exposure metrics.

## 2 Method

### 2.1 Processing System

**Fig. 1:**
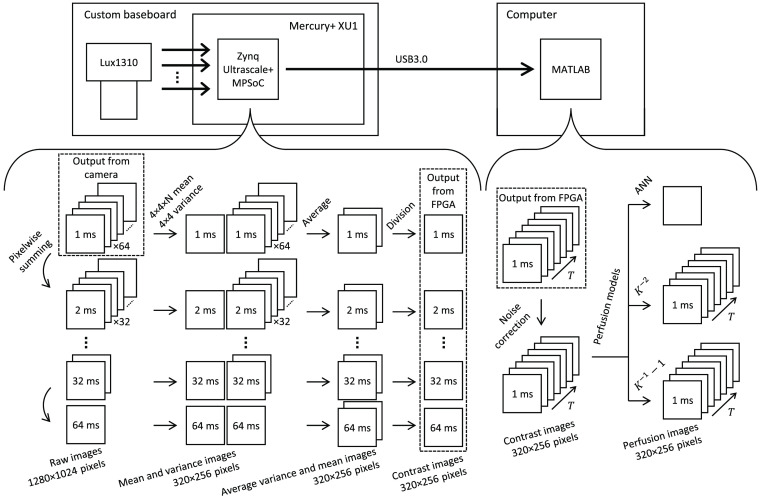
Visualization of the system flow from the Lux1310 camera sensor, through the multi-exposure contrast algorithm in the FPGA, and noise correction and perfusion models in the computer.

We designed and implemented an integrated system consisting of three main parts, as visualized in Fig. 1. We use a Lux1310 R2 CMOS camera sensor (Luxima Technology, Pasadena, California) capable of 1280×1024  pixels (1.3 megapixel), 12-bit images at 1000 fps. This connects to a Mercury+ XU1 SoC (Enclustra GmbH, Zürich, Switzerland; SoC—system on chip), equipped with a Zynq Ultrascale+ 15EG MPSoC (Xilinx, San Jose, California; MPSoC—multi-processing system on chip) and a 4-GB DDR4 SDRAM. The MPSoC contains both an FPGA and a 64-bit quad-core processor, enabling efficient implementation of the static image processing algorithm as well as dynamic tasks such as the transfer of data to the computer. The connection between the camera sensor and SoC is made by a custom-designed carrier board using 16 parallel low-voltage differential signaling (LVDS) data channels to enable continuous data transfers between the camera and SoC of 2  GB/s. The processed data from the SoC are sent via Universal Serial Bus (USB) 3.0 to a computer. This design with the custom carrier board and MPSoC addresses the problems we encountered in our previous iteration of the system^14^ where the transfer speed to the computer was insufficient.

The main portion of the MELSCI algorithm is implemented in the FPGA on the Zynq MPSoC. The implementation is functionally similar to our previous iteration,^14^ but for the benefit of the reader, we will explain the main ideas here as well. The algorithm takes a set of 64 consecutive images (1280×1024  pixels), each image with an exposure time of 998  μs and an interframe delay of 2  μs. For convenience, we refer to each image having an exposure time of 1 ms. Because the interframe delay is sufficiently small, we can create synthetic exposure times by pixelwise summing of pairs of images in this set, as first demonstrated by Dragojević et al.^13^ This doubles the exposure time for the summed images. Those can then be summed pairwise to again double the exposure time. This process continues until a single image with exposure time 64 ms remains. This results in seven different exposure times at powers of two, i.e., $T=2^{k}\;\mathrm{ms}$, k=0,1,…,6.

The number of images at each exposure time is N=64/T, for a total of 127 images. For each of these, we calculate the variance $\sigma_{n}^{2}(T)$ in nonoverlapping 4×4-pixel regions, reducing the image size by a factor of 16. For each exposure time, the average variance image $\sigma^{2}(T)=\frac{1}{N}\sum_{n=1}^{N}\sigma_{n}^{2}(T)$ is then calculated, where exposure time T is averaged over N images. We also calculate the average intensity ⟨I⟩ in the 4×4×64-pixel block, i.e., the average 1-ms intensity image. The average intensity for the other exposure times is calculated by scaling the 1-ms average intensity, i.e., ⟨I(T)⟩=T⟨I⟩. This is not an approximation, but an exact representation of the algorithm that allows it to be implemented more efficiently. Note that Fig. 1 depicts taking the average intensity separately for each exposure time. This is to make this figure easier to follow, and the result is equivalent for both methods.

In total, this process gives seven variance images, one for each exposure time, and one average image, and these are then used to calculate the squared speckle contrast

$$K_{\text{measured}}^{2}(T)=\frac{\sigma^{2}(T)}{T^{2}{\left\langle I\right\rangle }^{2}}.$$ (1)

All the calculations before this division are implemented in fixed-point format, and the division is performed by first converting the images to single-precision floating-point format. The precision loss due to this conversion is negligible. The seven $K^{2}(T)$ images are packaged together with the average image ⟨I⟩ as a 320×256×8-pixel multi-exposure contrast cube and sent to the computer over USB 3.0.

The entire processing chain is implemented in a pipelined fashion so that new images can be acquired while the previous set of 64 images is being processed. This enables continuous capture of images without any delay, for an effective frame rate of the output contrast cubes of 1000/64=15.6  fps.

### 2.2 Noise Correction and Calibration

Dark noise and shot noise in the raw images give rise to an unwanted increase in the contrast images.^11^ As has been described by others,^7^^,^^16^ in variance space, this can be modeled as a first-degree polynomial of the average intensity. For the exposure time used to capture images, T=1  ms, we get

$$\sigma_{\text{measured}}^{2}(1\;\mathrm{ms})=\sigma^{2}(1\;\mathrm{ms})+\langle I\rangle N_{\text{shot}}+N_{\text{dark}}+E_{\mathrm{const}},$$ (2)

where ⟨I⟩ is the average 1-ms intensity image, $\sigma^{2}$ is the variance due to speckle statistics, and $\sigma_{\text{measured}}^{2}$ is the variance we actually measure in the presence of noise. $N_{\text{shot}}$ and $N_{\text{dark}}$ describe the variance contribution from shot noise and dark noise, respectively, both of which are assumed to be Gaussian distributions. $E_{\mathrm{const}}$ describes the variance contribution from any constant inhomogeneities in the sensor, such as gain of individual pixels and different analog-to-digital converters. In a single-exposure system, $E_{\mathrm{const}}$ is indistinguishable from $N_{\text{dark}}$, but for multi-exposure they must be treated separately. In the ideal case, with a perfectly calibrated sensor, $E_{\mathrm{const}}$ will be zero. However, this is not always possible.

To correct the noise variance for longer exposure times, we could use Eq. (2) for each exposure time individually, effectively correcting seven independent single-exposure systems using two-parameter models (because $N_{\text{dark}}$ and $E_{\mathrm{const}}$ are indistinguishable in this case). However, under the assumption that the shot noise and dark noise in two consecutive 1-ms images are independent, we can extend Eq. (2) to a joint model for all exposure times T using the additivity of variances

$$\sigma_{\text{measured}}^{2}(T)=\sigma^{2}(T)+T\langle I\rangle N_{\text{shot}}+TN_{\text{dark}}+T^{2}E_{\mathrm{const}}.$$ (3)

Note the $T^{2}$ scaling for $E_{\mathrm{const}}$, which is due to the static pattern being identical from one image to the next, and therefore, the variance contribution grows with the square of the number of accumulated images T. We have validated this model on real data and in simulations (data not presented).

To estimate the constants $N_{\text{shot}}$, $N_{\text{dark}}$, and $E_{\mathrm{const}}$, we measured variance images over a wide range of intensities using a halogen light source (Flexilux 150 HL Universal, Schölly Fiberoptic GmbH, Denzlingen, Germany) inside an integrating sphere (barium sulfate coating, 10 cm diameter, Oriel Instruments, Stratford, Connecticut). The lens was removed to ensure a homogeneous illumination on the entire sensor, which was placed in the center of the opening in the sphere. We also equipped the sensor with a 785-nm bandpass filter (55-nm FWHM, BN785, Midwest Optical Systems Inc., Palatine, Illinois) to only characterize the noise at the laser wavelength. The dominant factor influencing the noise is the intensity of light impinging each pixel, thus the optical setup for this measurement could be different from normal use and still yield the correct noise constants. The temperature is also a factor that influences the amount of dark noise, but we avoided this issue by allowing sufficient warm-up time, ensuring similar temperatures for every measurement.

The intensity level was adjusted in 24 steps spanning a sufficiently large portion of the dynamic range of the sensor. For each intensity level, we measured 150 contrast cubes ∼9.6  s), which were converted to variance by use of Eq. (1). We then calculated the global spatiotemporal average for each exposure time and for the average intensity image. Equation (3) was fitted to the spatiotemporal averages of variances relative intensity using the function “fminsearch” in MATLAB 2020a (MathWorks, Natick, MA, USA). Since the light source used was not coherent and we ensured an even illumination, we could assume $\sigma^{2}(T)=0$ and thus estimate the noise constants $N_{\text{shot}}$, $N_{\text{dark}}$, and $E_{\mathrm{const}}$. These were then used in contrast space to make the correction according to

$$K_{\text{corrected}}^{2}(T)=K_{\text{measured}}^{2}(T)-\frac{N_{\text{shot}}}{T\langle I\rangle }-\frac{N_{\text{dark}}}{T{\left\langle I\right\rangle }^{2}}-\frac{E_{\mathrm{const}}}{{\left\langle I\right\rangle }^{2}},$$ (4)

where $K_{\text{corrected}}^{2}(T)$ is the noise corrected contrast and $K_{\text{measured}}^{2}(T)$ is the contrast output from the FPGA.

As a final calibration, we calculated the maximum contrast from measurements of a static homogeneous object illuminated by the laser source. The static object was a diffusely scattering and absorbing custom-made large-form factor ($20\times 20\times 2.5\;{\mathrm{cm}}^{3}$) optical phantom. Polydimethylsiloxane was used as scatterer and India ink as absorber. From this, we scaled the contrast to be in the range from 0 to 1 by

$$K^{2}(T)=\frac{K_{\text{corrected}}^{2}(T)}{K_{\max}^{2}(1\;\mathrm{ms})},$$ (5)

where $K_{\text{corrected}}^{2}(T)$ is from Eq. (4) and $K_{\max}^{2}(1\;\mathrm{ms})$ is calculated as the average from a large number of contrast images of the static object. Since $K_{\max}^{2}$ was measured on a static object, the decrease in contrast with exposure time was very small, and $K_{\max}^{2}(1\;\mathrm{ms})$ is, therefore, a good approximation of $K_{\max}^{2}(0\;\mathrm{ms})$. This effectively approximates the β-parameter used in many speckle contrast models, e.g., in Refs. 5, 8, and 13, which is a correction factor that accounts for the effect on contrast from having several speckles per pixel as well as from polarization.^8^ One could argue to correct each exposure time independently, but we choose to correct all exposures with the maximum contrast of the 1-ms image, not to introduce empirical nonlinearities in the processing.

The calibration was performed only once as part of the characterization of the sensor. It is reasonable to assume that the calibration constants will not change from one measurement to the next, as it depends only on the intensity and temperature and the latter was the same for all measurements.

### 2.3 Perfusion Estimation

After noise correction and calibration according to Eqs. (4) and (5), we estimated perfusion from the multi-exposure contrast in each pixel using a technique we have presented previously.^6^ Using 100,000 randomized tissue models spanning a wide range of properties such as blood flow speed distribution, blood amount, skin layer thickness, etc., together with Monte Carlo simulations of the light transport in these models, we created a large dataset from which we calculated both multi-exposure contrast and laser Doppler perfusion. We then trained an ANN to estimate laser Doppler perfusion from the multi-exposure contrast in each pixel, i.e.

$$P_{\mathrm{ANN}}=ANN[K^{2}(1\;\mathrm{ms}),K^{2}(2\;\mathrm{ms}),K^{2}(4\;\mathrm{ms}),\ldots ,K^{2}(64\;\mathrm{ms})],$$ (6)

where “ANN” represents the trained neural network. As we have shown previously, this technique achieves $R^{2}=0.990$ correlation between expected and estimated laser Doppler perfusion *in vivo* and is orders of magnitude faster than other available multi-exposure algorithms for estimating perfusion.^6^ This enables real-time calculation of perfusion images at the full 15.6 fps.

For comparison, we also calculated two single-exposure LSCI perfusion estimates at various exposure times. A common single-exposure model is the speckle flow index, the reciprocal of the speckle decorrelation time τ. There is no closed form solution for calculating τ from the speckle contrast and although it is possible to approximate the solution, for example, by analytic series expansion,^17^ the problem is often replaced with a simplified expression.^18^ This is directly proportional to the inverse of squared contrast, the same model that is used in the Moor FLIP-2 commercial device (Moor Instruments, Millwey, UK). Although they call the metric “flux,” we refer to this as perfusion

$$P_{K^{2}}(T)=\frac{1}{K^{2}(T)},$$ (7)

where T is the exposure time of the camera and K(T) is the speckle contrast at said exposure time. It is common to include a calibration factor in the model to change the scale of the perfusion values, but for the analysis in this paper this would yield equivalent results and is, therefore, omitted.

In addition to this, we compared with the perfusion model used in the Pericam PSI (Perimed AB, Järfälla, Stockholm, Sweden), another commercially available LSCI device

$$P_{K}(T)=\frac{1}{K(T)}-1.$$ (8)

One noteworthy difference between the single-exposure perfusion models is that the latter equals zero for K=1, which is expected when measuring on an object not containing any moving scatterers. Also note that the two single-exposure perfusion models utilize the same amount of recorded data as the MELSCI model when calculated in this study. For example, for an exposure time of 8 ms, the contrast is calculated as the average during 64 ms, i.e., from eight contrast values.

To compare the accuracy of the perfusion models, we used probe-based LDF as a reference technique. This estimates the laser Doppler perfusion $P_{\mathrm{LDF}}$, which compared to LSCI has a better understood theoretical basis and responds linearly to flow speeds.^9^^,^^10^ The process of calculating $P_{\mathrm{LDF}}$ is not the focus of this paper but has been described elsewhere.^5^

### 2.4 Measurements and Optical Setup

The camera was equipped with a 12-mm lens (Kowa Optimed Deutschland GmbH, Düsseldorf, Germany) set to #f/1.4 (maximum aperture) and a 785-nm bandpass filter (55-nm FWHM, BN785, Midwest Optical Systems Inc., Palatine, Illinois). The focus distance was set at 35 cm, and all measurements were performed at 26 to 27 cm from the camera. This ensured that the image was out of focus, which reduced the influence of structural contrasts in the surface, increasing the contrast-to-noise ratio. The need for this is a consequence of the large aperture (see Sec. 4).

**Fig. 2:**
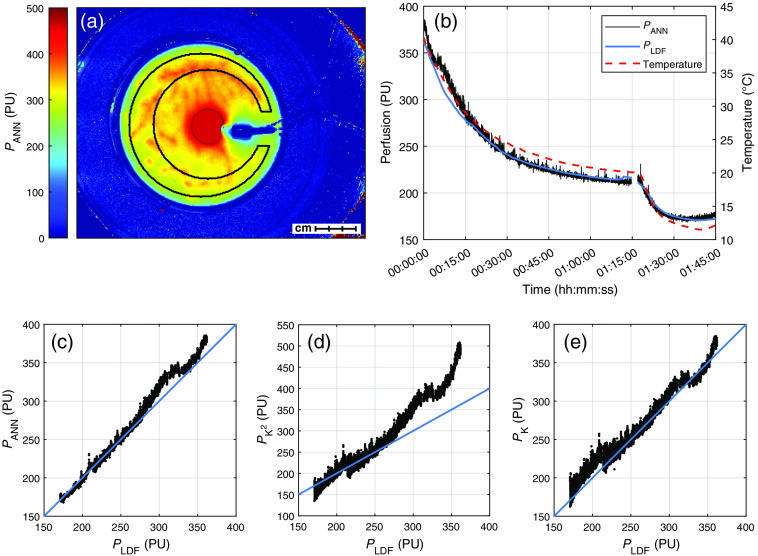
(a) Image of $P_{\mathrm{ANN}}$ at the beginning of the experiment, chosen at 4 min in (b). The ROI used to create (b)–(e) is also shown. (b) $P_{\mathrm{ANN}}$, $P_{\mathrm{LDF}}$, and temperature of the milk phantom as a function of time. (c)–(e) Regression plots of the three camera-based perfusion estimates against $P_{\mathrm{LDF}}$. The blue lines correspond to a perfect agreement with $P_{\mathrm{LDF}}$. The data were normalized to the average perfusion at 25°C.

To evaluate the perfusion estimate from the MELSCI system, we simultaneously measured $P_{\mathrm{ANN}}$, probe-based laser Doppler perfusion $P_{\mathrm{LDF}}$, and temperature in a beaker of milk (12 cm diameter, 1 cm deep). $P_{\mathrm{LDF}}$ and temperature were measured using a PF6010 LDPM/temp unit (Perimed AB). The temperature was varied from 50°C to 10°C using a water bath with hot water first cooled by ambient room temperature, then by ice [see Fig. 2(b)]. The milk beaker was raised 1 cm above the floor of the water bath, allowing homogeneous heating of the entire milk, thus eliminating most effects on the results due to delayed heating in the center. Due to Brownian motion, the measured perfusion in the milk increased with temperature.^19^ The thermistor and laser Doppler probe were fixed in place ∼2  mm below the surface of the milk in the center of the beaker. To avoid specular reflections, the camera was placed at an angle of ∼20  deg to the normal of the milk surface. This had no discernible effect on the perfusion image.

We also performed an occlusion-release provocation of the forearm of a healthy male subject, 47 years old. After the subject rested 15 min in a supine position in a temperature-controlled room (23°C), we measured 5-min baseline, 5-min brachial occlusion at 200 mmHg, and 5 min after releasing the occlusion. The measurement protocol was approved by the Regional Ethical Review Board in Linköping, Sweden (D. No. 2018/282-31), and written informed consent was obtained from the subject before the measurement.

## 3 Results

### 3.1 Validation Measurements

The results of the validation measurement are presented in Fig. 2. The perfusion estimates in (b)–(e) were averaged over the region of interest (ROI) shown in (a), except, of course, for $P_{\mathrm{LDF}}$ in (b). The laser Doppler signal was unaffected by the illumination from the MELSCI system, but the light from the laser Doppler probe interfered with the perfusion signal of the MELSCI system, as seen by the high perfusion in the center of the beaker. The ROI was selected to minimize this influence. The large ROI also removed some of the influence of slow convection movements in the milk, seen as the local variations in the image. To remove the influence of convection movements in $P_{\mathrm{LDF}}$, it was smoothed using a 10-min moving average filter. This also removed higher frequency noise that was due to the small sampling volume of the laser Doppler system. Furthermore, $P_{\mathrm{LDF}}$, $P_{K^{2}}(T)$, and $P_{K}(T)$ were scaled to the average value of $P_{\mathrm{ANN}}$ in the temperature range 24.5°C to 25.5°C. We can see in Fig. 2(b) that this corresponds to $P_{\mathrm{ANN}}\approx 250\;\mathrm{PU}$.

Figure 2(b) shows $P_{\mathrm{ANN}}$, $P_{\mathrm{LDF}}$, and temperature over time, where the temperature axis was scaled to show the high similarity to the perfusion. The bottom row of Fig. 2 presents $P_{\mathrm{ANN}}$, $P_{K^{2}}(T)$, and $P_{K}(T)$, all relative to $P_{\mathrm{LDF}}$ after normalization to 25°C. For this comparison, $P_{K^{2}}(T)$ and $P_{K}(T)$ were evaluated at 8 ms, but results for all exposure times are presented in Tables 1 and 2. The relation between $P_{\mathrm{ANN}}$ and $P_{\mathrm{LDF}}$ corresponds to a coefficient of determination of $R^{2}=0.992$, in line with our previous results using the ANN model.^6^ As summarized in Table 1, this value of $R^{2}$ is higher than $R^{2}$ for both single-exposure models at all exposure times. This indicates that $P_{\mathrm{ANN}}$ uses the extra information present in multiple exposure times to improve the ability of the model to resemble $P_{\mathrm{LDF}}$.

**Table 1:** $R^{2}$ of the camera-based perfusion estimates relative to $P_{\mathrm{LDF}}$. ME is for multi-exposure, using all 7 exposure times.

		Exposure time (ms)						
	ME	1	2	4	8	16	32	64
$P_{\mathrm{ANN}}$	0.992							
$P_{K^{2}}(T)$		0.975	0.972	0.971	0.969	0.966	0.960	0.935
$P_{K}(T)$		0.988	0.986	0.984	0.980	0.974	0.965	0.946

**Table 2:** Slope coefficients of a first-degree polynomial fit to each camera-based perfusion estimate relative to $P_{\mathrm{LDF}}$. Before fitting, the data were normalized to the average perfusion at 25°C. ME is for multi-exposure, using all 7 exposure times.

		Exposure time (ms)						
	ME	1	2	4	8	16	32	64
$P_{\mathrm{ANN}}$	1.090							
$P_{K^{2}}(T)$		1.118	1.373	1.512	1.541	1.483	1.356	1.173
$P_{K}(T)$		1.175	1.146	1.074	0.977	0.867	0.745	0.608

In Table 2, we also present the slope coefficients of the first-degree polynomials fit to each camera-based perfusion measure relative to $P_{\mathrm{LDF}}$ after normalization to 25°C. It is apparent that the slope is strongly dependent on the selected exposure time for $P_{K^{2}}(T)$ and $P_{K}(T)$.

### 3.2 In vivo Measurement

**Fig. 3:**
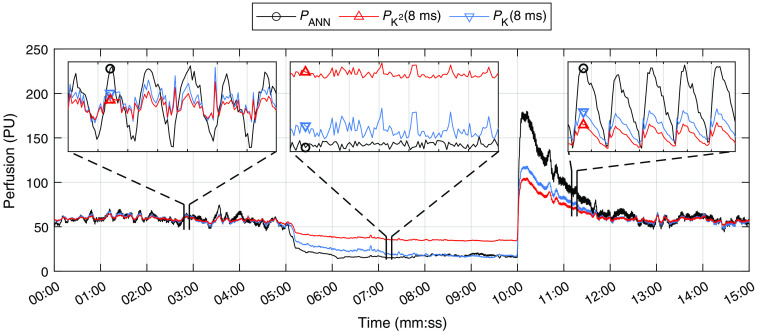
$P_{\mathrm{ANN}}$, $P_{K^{2}}(8\;\mathrm{ms})$, and $P_{K}(8\;\mathrm{ms})$ during an occlusion-release provocation of the right forearm of a healthy subject. Data on the full timescale have been averaged with a 2-s moving window to remove heart beats. The data in the inlays were not averaged and show a clear heartbeat signal during baseline and reperfusion; gridlines in the inlay plots indicate 1-s intervals. $P_{K^{2}}$ and $P_{K}$ were normalized to have the same baseline average as $P_{\mathrm{ANN}}$ (0:00 to 5:00 min).

**Fig. 4:**
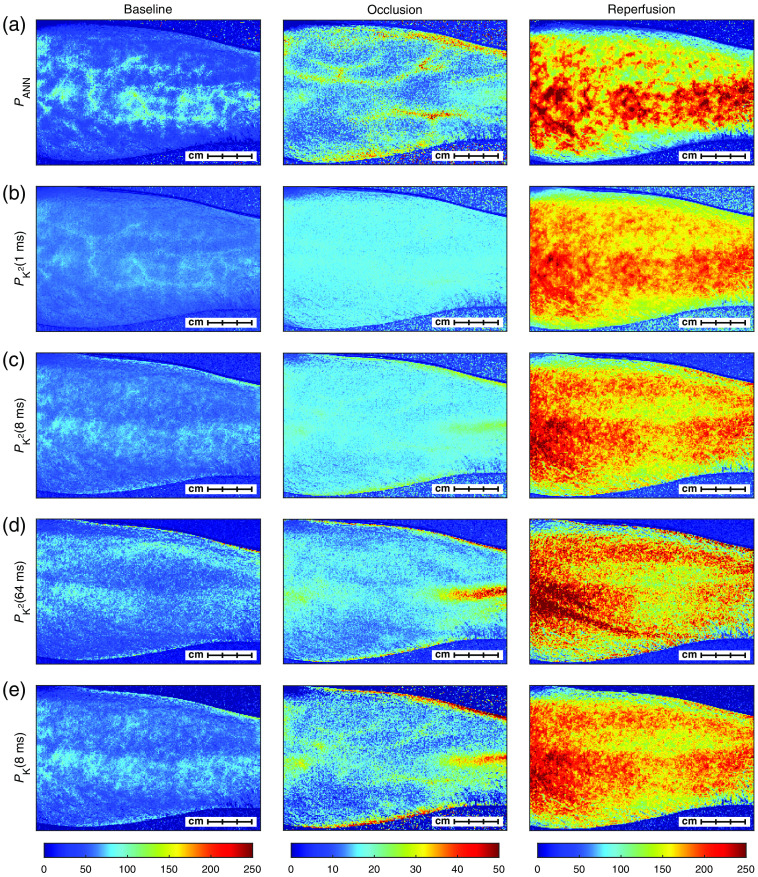
(a) Images of $P_{\mathrm{ANN}}$; (b)–(d) images of $P_{K^{2}}(1\;\mathrm{ms})$, $P_{K^{2}}(8\;\mathrm{ms})$, and $P_{K^{2}}(64\;\mathrm{ms})$; and (e) images of $P_{K}(8\;\mathrm{ms})$ at the three phases of the occlusion-release provocation. The columns correspond to baseline (selected at 4:00), occlusion (selected at 8:00), and reperfusion (selected at 10:06), respectively. Each image was averaged over 2 s (31 images). The $P_{K^{2}}$ and $P_{K}$ images have been individually scaled to match the average perfusion of the corresponding $P_{\mathrm{ANN}}$ image in a large ROI. This highlights the structural differences between the three techniques rather than the absolute values of the perfusion estimates. Note that the color scale for the occlusion phase is different from the other phases to make the vascular structures more prominent (Video S1, MP4, 45.8 MB [URL: https://doi.org/10.1117/1.JBO.25.11.116007.1]).

The results from the *in vivo* occlusion-release experiment are presented in Figs. 3 and 4. In Fig. 3, $P_{\mathrm{ANN}}$ is compared with $P_{K^{2}}(8\;\mathrm{ms})$ and $P_{K}(8\;\mathrm{ms})$ in a large ROI covering most of the visible area of the arm in Fig. 4. The single-exposure perfusion estimates $P_{K^{2}}$ and $P_{K}$ were normalized to $P_{\mathrm{ANN}}$ to give the same average baseline (0:00 to 5:00 min). A 2-s moving average window was applied to the data on the full timescale to remove the heartbeat signal. The data in the zoom plots show the full-time resolution without the 2-s averaging, i.e., including the heartbeats. The gridlines in the zoom plots indicate 1-s intervals, and the range of perfusion values in the zoom boxes are indicated by the height of the corresponding selection lines. One might think there is a discrepancy between the reperfusion data in the full timescale and the zoom, where the zoomed data are overlapping but this is not reflected in the selected data. However, this is a side effect of the 2-s time average and not an error in the data.

$P_{\mathrm{ANN}}$ displays higher dynamics during both baseline and reperfusion and shows significantly less noise than the two single-exposure estimates (most easily seen in the inlay baseline plot). Both factors contribute to a more clearly visible heartbeat signal in baseline and reperfusion. The reperfusion peak is also significantly higher for $P_{\mathrm{ANN}}$ than for the two other estimates.

Figure 4 compares images of $P_{\mathrm{ANN}}$ with $P_{K^{2}}$ at 1, 8, and 64 ms exposure time and with $P_{K}$ at 8-ms exposure time during baseline, occlusion, and reperfusion. Note that the color scale is different for occlusion to enhance the vascular structure in those images. All images were calculated as time averages over 2 s (31 images) at time 4:00 min (baseline), 8:00 min (occlusion), and 10:06 min (reperfusion). To make visual comparison easier, all $P_{K^{2}}$ images and $P_{K}$ images were scaled to have the same average perfusion of the corresponding $P_{\mathrm{ANN}}$ image in a large ROI covering most of the arm. This means that the relation between $P_{K^{2}}$ and $P_{K}$ images at baseline, occlusion, and reperfusion is not representative of the exact change in perfusion, but it enhances structural differences between the different exposure times and $P_{\mathrm{ANN}}$. Interestingly, while $P_{\mathrm{ANN}}$ is most similar to $P_{K^{2}}(1\;\mathrm{ms})$ for baseline and reperfusion, it resembles $P_{K^{2}}(8\;\mathrm{ms})$ more during occlusion. This indicates that the ANN model utilizes the multi-exposure information to extend the dynamic range of the perfusion estimate.

The results from the occlusion-release provocation are also presented in Video S1. It shows a playback of the experiment with the $P_{\mathrm{ANN}}$ time trace corresponding to Fig. 3 and image corresponding to Fig. 4. A slight difference is that we applied a 0.5-s moving average instead of 2 s, allowing the heartbeat signal to be visible in the video.

## 4 Discussion

In this study, we have shown the first complete implementation of an MELSCI system capable of continuous real-time video-rate acquisition and processing. We compared the perfusion estimate from our machine learning model to two commonly used single-exposure metrics. With these results, we have shown how perfusion imaging can gain the benefits of more information from multi-exposure while retaining the speed of single-exposure systems.

The system presented in this paper is similar to our previous system.^14^ However, there are some important differences. The previous design implemented the contrast algorithm with sufficiently high data throughput, whereas the transfer of processed data to the computer was very slow. This was a consequence of utilizing a pure FPGA-based chip in that design. We, therefore, suggested that an SoC with both an FPGA and a hard central processing unit (CPU) could address this issue. Hence, we designed a new system based on the Zynq Ultrascale+ MPSoC, which solves this bottleneck. Furthermore, when we presented the previous system, we had not yet developed an algorithm capable of converting multi-exposure data to perfusion images in real-time, thus the ANN model was a necessity for the results in this paper. Despite the faster implementation, it is still worth noting that we have to use nonoverlapping pixel regions in the contrast calculation to reduce the amount of data to a manageable amount that can be transferred to the computer. This also reduces the resolution of the perfusion image, which is why it is common to use a sliding pixel region to retain the original image resolution. In this work, we valued the video-rate frame rate higher than the image resolution. However, in the future, if the ANN can be implemented in the FPGA, the reduction in output data would allow us to instead use a sliding window and thus retain the original image resolution.

The implementation of the algorithm and real-time perfusion model is, in principle, independent of the optical setup, and the discussion of the two should be held separate. However, it is worth noting that using an f-number of 1.4 will cause many speckles per pixel. Although the correction using $K_{\max}^{2}$ according to Eq. (5) has been validated previously,^6^^,^^20^ too many speckles per pixel will increase the relative influence of nonspeckle related image contrast from the tissue surface, e.g., from wrinkles and hair. This is a trade-off, since decreasing the aperture also decreases the signal-to-noise ratio.^6^^,^^21^ Due to the short-exposure time, we have determined that having the maximum amount of light is beneficial, but this is an area of possible improvement. We mitigate some of the effect of surface contrasts by measuring out of focus.

We choose not to compare $P_{\mathrm{ANN}}$ with any other multi-exposure perfusion model in this study. As far as we are aware there are currently no other multi-exposure models that can run in real-time at sufficiently high frame rates. Only single-exposure models have had that capability, which is why we consider those comparisons are more appropriate for this paper. We have previously compared $P_{\mathrm{ANN}}$ with another multi-exposure model in an offline setting and shown that our model has a higher correlation to laser Doppler perfusion and is less susceptible to measurement noise.^6^ For the single-exposure data, we mostly chose the 8-ms data because that is closest to what is used in commercial devices. We argue this is relevant since it shows the possible improvement in accuracy over most clinical studies that have been done with those devices.

As seen in Table 1, the results from the milk phantom in Fig. 2 show a higher $R^{2}$ to laser Doppler perfusion for $P_{\mathrm{ANN}}$ compared to $P_{K^{2}}$ and $P_{K}$, even with an optimized exposure time. This suggests that $P_{\mathrm{ANN}}$ uses the information present in multiple exposure times to improve the perfusion estimate, both in terms of linearity to laser Doppler perfusion and noise. In Figs. 2(c)–2(e), some similarities can be seen between the regression plots of $P_{\mathrm{ANN}}$, $P_{K^{2}}$, and $P_{K}$, which is not surprising considering they use the same contrast to calculate the respective perfusions. Some of the deviation in the linearity between $P_{\mathrm{LDF}}$ and the camera-based perfusion estimates can be explained by the fact that $P_{\mathrm{LDF}}$ is measured in a single point that may not correlate entirely with the perfusion in the imaged sampling volume. Temperature gradients due to rapid heating or cooling of the water bath can also explain some of the deviations. Furthermore, these temperature gradients cause convection movements in the milk. These appear as inhomogeneities in Fig. 2(a) and become obvious when viewed in video form. The perfusion is probably influenced by this in the beginning of the experiment. The convection movements eventually settle down around t=15  min in Fig. 2(b), when the temperature has decreased to 30°C. If we limit the analysis to t=15  min and after, the slope coefficients in Table 2 become closer to 1 for all exposure times for $P_{K^{2}}(T)$, and for T≤4  ms for $P_{K}(T)$. For $P_{\mathrm{ANN}}$, the slope essentially becomes equal to 1.

The time traces in Fig. 3 show higher dynamics in $P_{\mathrm{ANN}}$ compared to the single-exposure models, where the relative increase in the reperfusion peak compared to end of occlusion is ∼3 times for $P_{K^{2}}$, 7 times for $P_{K}$, and 12 times for $P_{\mathrm{ANN}}$. This indicates that $P_{\mathrm{ANN}}$ is more sensitive to higher flow speeds than the two single-exposure models. This is expected as $P_{\mathrm{ANN}}$ is more linear with respect to $P_{\mathrm{LDF}}$ than $P_{K^{2}}$ and $P_{K}$, and $P_{\mathrm{LDF}}$ is, in turn, linear with respect to flow speed.^5^ As demonstrated by Thompson and Andrews,^12^ MELSCI data from a sufficiently wide range of exposure times can be used to directly calculate the Doppler spectra. It is, therefore, reasonable to assume that $P_{\mathrm{ANN}}$ is sensitive to the same changes in the Doppler spectra as $P_{\mathrm{LDF}}$. $P_{\mathrm{LDF}}$ is linear to speed because it is linear to the average Doppler frequency shift, which is linear to speed.^22^ Multiple Doppler shifts also cause a broadening of the Doppler spectrum, suggesting that $P_{\mathrm{ANN}}$ is more sensitive to multiple Doppler shifts than $P_{K^{2}}$ and $P_{K}$ for the same reason that it is more sensitive to speed changes. Multiple Doppler shifts are more common at higher blood concentrations and have a larger influence on light that has penetrated deeper into the tissue. We, therefore, hypothesize that the depth in tissue at which the models can extract information is larger for $P_{\mathrm{ANN}}$ than for $P_{K^{2}}$ and $P_{K}$, i.e., it is more sensitive to light that has penetrated deeper and been multiple Doppler shifted. This may be another reason that the increase in the reperfusion peak is larger for $P_{\mathrm{ANN}}$.

In Fig. 3, it can also be seen that $P_{\mathrm{ANN}}$ is less noisy than the other two signals, which is likely because the ANN has been trained to recognize measurement noise and account for it in the perfusion estimate. While we did not bring up the details of the ANN training in this paper, the full explanation of the process has been discussed previously.^6^ The difference in noise is also evident in the milk phantom measurements in Fig. 2.

Figure 4 indicates that $P_{\mathrm{ANN}}$ is more sensitive to larger vascular structures than the two single-exposure models. During baseline and especially reperfusion, there are well-defined hotspots and coldspots in the perfusion images that persist over time. This can be seen more clearly in the video rendering of the experiment (Video S1). While these spatial heterogeneities in the perfusion are present in the other images, especially in the $P_{K^{2}}(1\;\mathrm{ms})$ image, they are less visible and more diffuse than for $P_{\mathrm{ANN}}$. It is also apparent that the variations during the heart cycle are much larger in $P_{\mathrm{ANN}}$ than in the two single-exposure models. This increased spatial and temporal variability could be a further indication that $P_{\mathrm{ANN}}$ is more sensitive to deeper structures in the skin where flow is generally higher, as discussed before.

It may seem surprising that the vasculature of some large vessels can be seen during the arterial occlusion, especially for $P_{\mathrm{ANN}}$, but also for 8-ms exposure time for the other perfusion measures. This is likely an effect of the process where the vessels on the arterial side are dilating in response to the reduced oxygen saturation, leading to a redistribution, i.e., movement, of the blood. The result of this redistribution can be seen during arterial occlusion when measuring with diffuse reflectance spectroscopy, where the amount of blood in the skin increases during the occlusion.^23^ It is interesting to see that for single-exposure LSCI, the vasculature is more pronounced in $P_{K^{2}}(1\;\mathrm{ms})$ than in the other exposure times during baseline, but more in $P_{K^{2}}(8\;\mathrm{ms})$ during the occlusion, whereas it is even more pronounced in $P_{\mathrm{ANN}}$ during both baseline and occlusion. This is yet another indication that the multi-exposure data processing can handle information from different exposure times in a manner that results in valuable information about the underlying flow. It is also an indication that the measurement depth is larger, as discussed above.

We expect that this new technique can be applied clinically in any setting where single-exposure LSCI has been used, while also providing higher quality data. This is especially true in applications where simultaneous assessment of spatial and temporal dynamics is needed. A few such areas where LSCI has been used are burn wounds,^24^ intraoperative monitoring of cerebral blood flow,^25^ and stroke models,^26^ where the function of the microcirculation can vary a lot for different tissue regions. In a stroke animal model, Parthasarathy et al.^27^ showed that MELSCI is more accurate than single-exposure LSCI, partially due to the ability to estimate flow in the presence of static scatterers. Another example where the spatial information is important is in patients with sepsis, where a significant spatial heterogeneity can be observed in the microcirculation.^28^ It has also been suggested that LSCI could become a useful tool for monitoring diabetic foot ulcers,^29^ where most studies to date have used laser Doppler perfusion imaging,^30^ and, therefore, the MELSCI system that we have suggested may be important due to its LDF mimicking properties. The increased quality in the pulsatile signal in $P_{\mathrm{ANN}}$ compared to the single-exposure models is also interesting. Ghijsen et al.^31^ found that the speckle signal in transmission through a finger had a higher frequency content in younger subjects that in older, proposing this to be related to arterial stiffening. Although that study utilized much deeper measurements than in this study, we see the possibility of performing a similar analysis to get spatial maps of pulsatile information, which might enable new markers of tissue microcirculatory function.

## 5 Conclusion

Since Parthasarathy et al.^11^ first presented the idea of MELSCI, the limitation has always been in the amount of data that is required for the technique to be effective. Several reviews of the field have concluded that more work should be put into developing the systems and algorithms for MELSCI.^4^^,^^8^ As far as we are aware, this paper presents the first MELSCI system to successfully run continuously in real-time with a video-rate speed comparable to those seen in commercial single-exposure systems, while enabling more sophisticated and powerful models that can use the multi-exposure information for a more accurate perfusion estimate. We believe this is an important step forward in making LSCI feasible for clinical use.

## 6 Appendix A—Video S1

Video S1 shows an arterial occlusion-release provocation of the forearm of a healthy male subject, 47 years old. The provocation protocol was a 5-min baseline, followed by a 5-min brachial occlusion at 200 mmHg, and 5 min after release of the occlusion. The image and graphs show MELSCI perfusion $P_{\mathrm{ANN}}$, where the graphs are averaged in the ROI marked in the image. A 0.5-s moving average is applied to the images and full timescale plot. The graph in the 20-s plot is not averaged and shows the full temporal resolution. The video is fast forwarded to interesting time points.
